# Therapeutic Potential of Triptolide in Treating Bone-Related Disorders

**DOI:** 10.3389/fphar.2022.905576

**Published:** 2022-06-15

**Authors:** Wu Gang, Hu Hao, Huang Yong, Feng Ruibing, Li Chaowen, Huang Yizheng, Li Chao, Zhang Haitao

**Affiliations:** ^1^ Department of Spinal Surgery, Hubei Provincial Hospital of TCM, Wuhan, China; ^2^ Affiliated Hospital of Hubei University of Chinese Medicine, Wuhan, China; ^3^ Hubei Provincial Academy of Traditional Chinese Medicine, Wuhan, China; ^4^ Wuhan Sports University, Wuhan, China

**Keywords:** *Tripterygium wilfordii* Hook. F., triptolide, osteosarcoma, inflammatory disorders, bone diseases, molecular mechanism

## Abstract

Triptolide, a diterpene triepoxide, is a pharmacologically active compound isolated from a Chinese medicinal herb *Tripterygium wilfordii* Hook F (TwHF). Triptolide has attracted considerable attention in recent times due to its multiple biological and pharmaceutical activities, with an emphasis on therapeutic importance in the treatment of diverse disorders. With essential medicinal implications, TwHF’s extracts have been used as anti-inflammatory, antiproliferative, antioxidative, and immunosuppressive agents for centuries, with continuous and relevant modifications to date to enhance its utility in several diseases and pathophysiology. Here, in this review, we accentuate the studies, highlighting the effects of triptolide on treating bone-related disorders, both inflammatory and cancerous, particularly osteosarcoma, and their manifestations. Based on this review, future avenues could be estimated for potential research strategies, molecular mechanisms, and outcomes that might contribute toward reinforcing new dimensions in the clinical application of triptolide in treating bone-related disorders.

## Introduction

**FIGURE 1:**
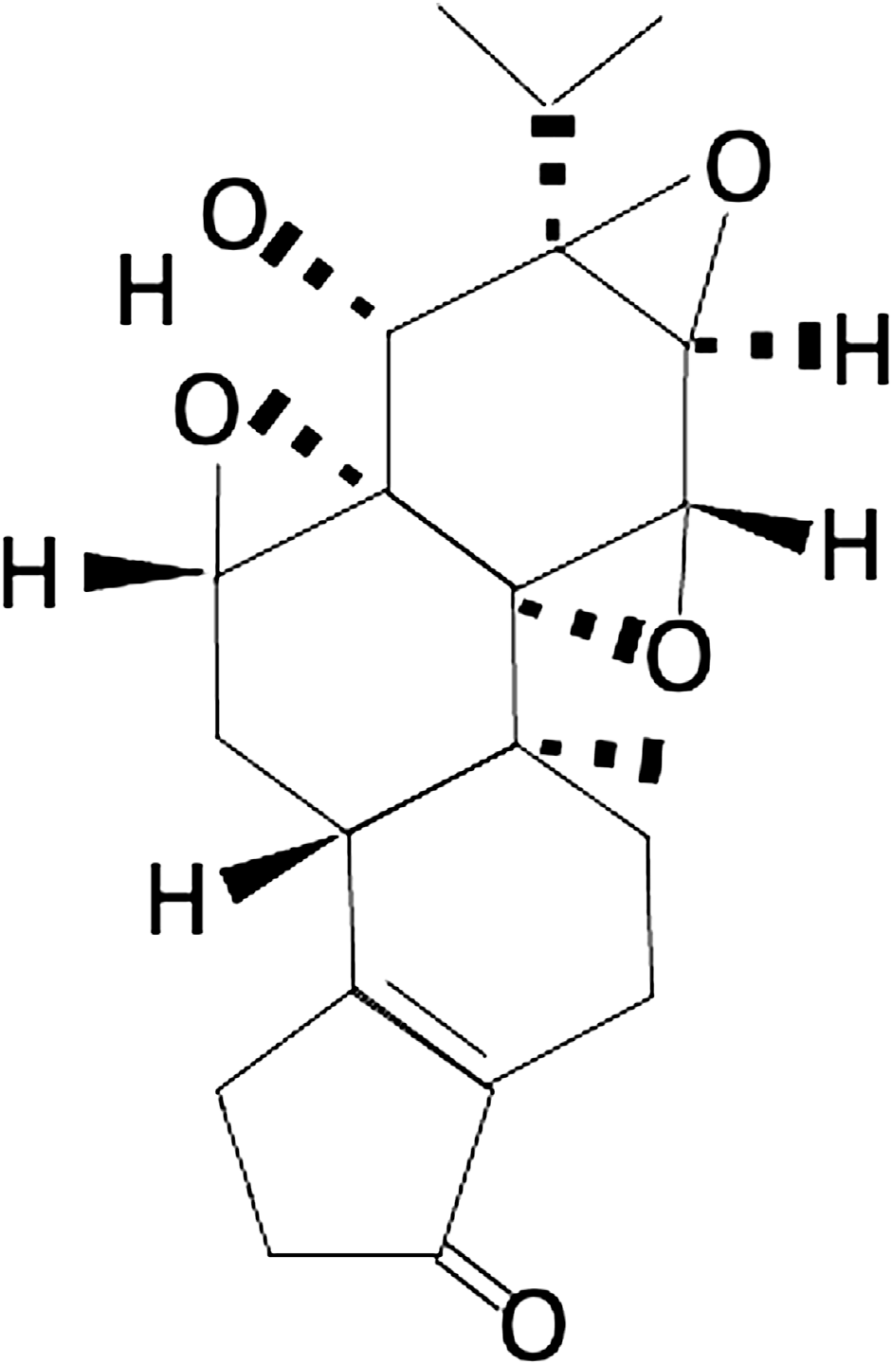
Chemical structure of triptolide: triptolide has been shown to possess a broad spectrum of anti-inflammatory, anti-immunosuppressive, and anti-oxidative properties in treating various disorders.

With a history of about 3,000 years, traditional Chinese medicine (TCM) continues to get the attention of the academic and industrial world. However, it was only in the 20th century that TCM was revisited through modern science technologies. Being an essential part of the Chinese healthcare system, TCM has been recognized by the WHO. Thereafter, the International Classification of Diseases (ICD 11) has introduced a new chapter on traditional medicine. TCM is also widely used in the West and known as complementary or alternative medicine (Gu and Pei, 2017; Xu and Yang, 2009). Chinese herbal medicines (CHMs), a subdiscipline and an essential part of TCM, include single herbs, Chinese proprietary medicines, and mixtures of different herbs, which might be used alone or in combination with Western medicines. CHMs have been demonstrated to cure a wide variety of ailments, from simple to complicated and acute to chronic conditions (Gu and Pei, 2017; Xu and Yang, 2009). Nowadays, from the drug discovery aspect, screening of functional compounds designated in the list/guidelines under the field of CHM (a subdiscipline branch of TCM) as herbal medicines is more efficient than random screening of a synthetic combinatorial chemical library (Xu and Liu, 2019). The *Tripterygium wilfordii* extract contains bioactive components, such as triptolide, celastrol, and tripchlorolide. Nuclear magnetic resonance (NMR) and mass spectroscopy have characterized more than 46 diterpenoids (e.g., triptolide), 20 new triterpenoids (e.g., celastrol), 21 alkaloids (e.g., euonine), and other small molecules from TwHF, which possess pharmaceutic and medicative importance (Wong et al., 2012). Herbal extracts from this plant have been widely used in the treatment of autoimmune and inflammatory diseases, including rheumatoid arthritis (RA). Triptolide (Figure 1), a major component of TwHF, has many beneficial functions, including immunosuppression, anti-inflammation, and anticarcinogenesis. The pharmacological influence of triptolide towards clinical progress showcases its role in different anti-inflammatory, immunomodulatory, antioxidant, and antiproliferation activities (Tang et al., 2020; Tong et al., 2021; Yu et al., 2021; Yang et al., 2022). Furthermore, triptolide has been reported to play a critical role in various maladies, such as neurological disorders, respiratory illness, gastrointestinal disorders, renal diseases, endocrine diseases, autoimmune disorders, bone dysfunction, and cancer (Yuan et al., 2019; Cheng et al., 2021). Herein, we focused on featuring the effects of chemical constituents, bioactivity, and therapeutic window of triptolide on treating bone defects and its related manifestations in inflammations and cancer, especially on bone cancer such as osteosarcoma.

## Taxonomical Narration

*Tripterygium wilfordii* Hook F (TwHF) is a traditional Chinese medicinal herb, commonly known as “Thunder God Vine” or “Lei Gong Teng.”. *Tripterygium wilfordii*, a perennial woody vine member of the Celastraceae family, is native to eastern and southern China, Korea, and Japan. It has been used as a traditional and allopathic Chinese herb for centuries (Brinker et al., 2007). *Tripterygium* has four species: T. *wilfordii* Hook F, *Tripterygium* spp., *T. hypoglaucum* (Lévl.) Hutch, and *T. regelii* Sprague & Takeda. T. *hypoglaucum* (Lévl.) Hutch shares its name with *Aspidopterys hypoglauca* H. Lév, *T*. *forrestii* Loes, and *T*. *wilfordii* var. exesum Sprague & Takeda (https://www.ars-grin.gov/). *T*. *wilfordii* and *T*. *hypoglaucum* are conspecific, while *T*. *regelii* likely constitutes a separate species. The results of the phylogeographic and phylogenetic analysis by Ma et al. (2017) have demonstrated that *T*. *hypoglaucum* and *T*. *wilfordii* are clustered together in phylogenetic trees, the haplotype network, and a spatial analysis of molecular variance (SAMOVA) and therefore suggested that *T*. *wilfordii* and *T*. *hypoglaucum* could be considered a single species with the name *T*. *wilfordii* Hook F while *T*. *regelii* is a separate species with the name *T*. *regelii* Sprague et Takeda.

## Traditional Medicinal Importance

Herbal products always receive utmost attention due to their wide range of usage in traditional medicinal systems, regardless of whether it is Chinese, Indian, Egyptian, or Greek. A wide range of herbal preparations either in the whole crude drug or in their purified components has shown to be beneficial both in *in vivo* and *in vitro* experimental studies. As a result, such herbal medicines or their active components have been credited to be indispensable sources to design modern drugs and have garnered widespread attention to serve as an invaluable source of some widely used medicines. However, their clinical potential remains to be fully explored. Over 400 natural products have been isolated and characterized from TwHF, which include triptolide, wilforcidine, wilfordine, and celastrol. Among all the active components, triptolide has been studied and explored widely. Triptolide was identified by Kupchan in 1972 (Tong et al., 2021), and it exhibited almost all the therapeutic activities of TwHF extracts, with the toxic content as high as that of TwHF. As stated earlier, triptolide has been extensively studied for its mechanism of action, pharmacological activity, and toxicity. So far, several pharmacological aspects of this active constituent have been reported by researchers in different pathologies. For information about the structure and advanced triptolide medicinal chemistry, comprehensive articles could be referred (Hou et al., 2019; Tong et al., 2021). TwHF extracts were assessed in clinical studies for treating a variety of diseases, including cancer (Noel et al., 2019), rheumatoid arthritis (Fan et al., 2016; Fan et al., 2018), ankylosing spondylitis (Wang et al., 2018), Crohn’s disease (Chen et al., 2019), systemic lupus erythematosus (SLE) (Xiao et al., 2022), kidney transplantation (Fan et al., 2016), and a variety of skin diseases (Qi et al., 2021).

## Synchronization of Triptolide and Bone-Related Diseases

**FIGURE 2:**
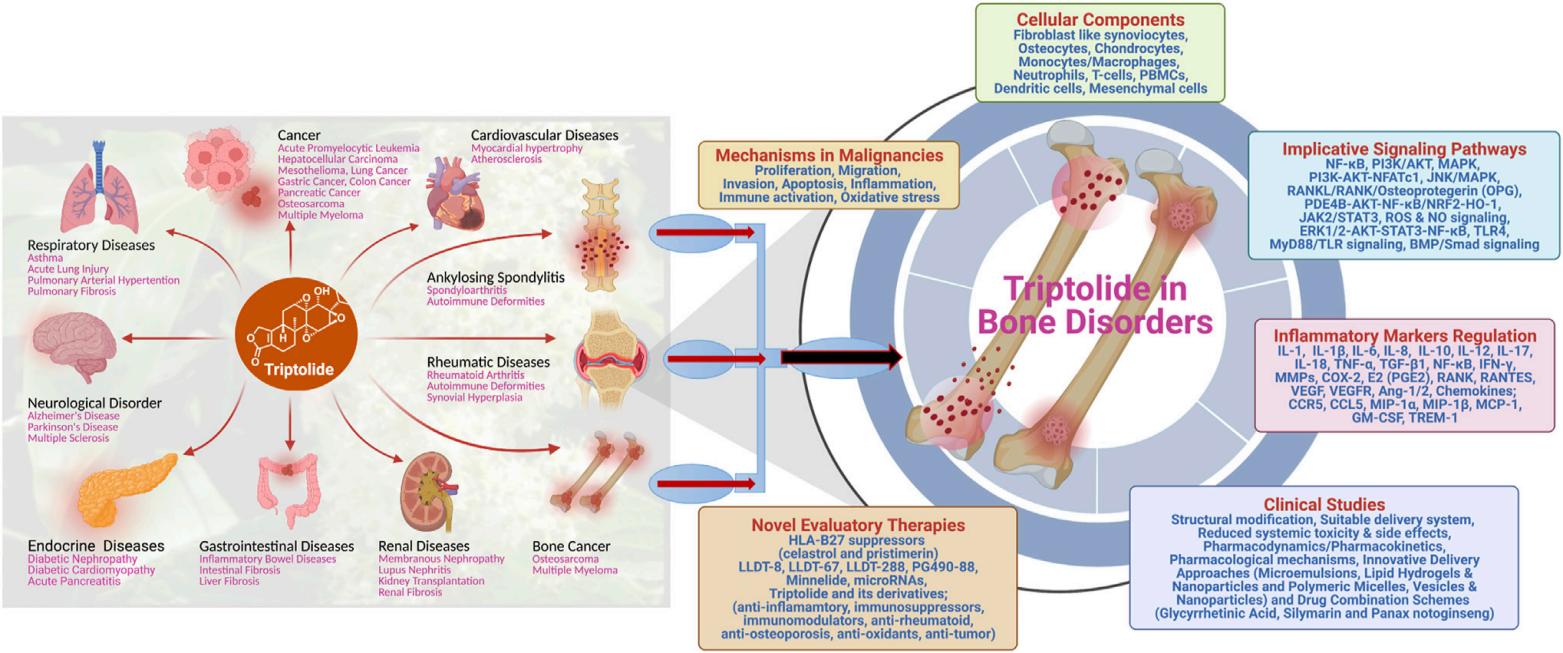
Schematic illustration of the multifaceted role of triptolide in treating different diseases, with exclusive therapeutic potential in treating bone disorders.

Bone-related disorders consist of both bone-disease types and joint-disease types that broadly encompass conditions such as osteoporosis, osteomalacia, metabolic bone dysfunction, hyperparathyroidism, Paget disease, fracture/stress fracture, bone cancer, scoliosis, osteoarthritis, rheumatoid arthritis, spondyloarthritis, juvenile idiopathic arthritis, lupus, gout, and bursitis (Terkawi et al., 2022; Wu et al., 2022; Young et al., 2022) [https://health.usnews.com/conditions/bone-and-joint-disease]. These diseases are multisymptomatic, and their treatment dose is largely varied depending on the medicative adaptability of the regimen (Akkoc and Khan, 2016; Avdic et al., 2006; Balakrishnan and Madnani, 2013; Toussirot and Wendling, 1996; van der Linden et al., 2015). Clinical implications of triptolide in bone-related pathologies have increased dramatically in the recent past. Several molecular mechanisms have been proposed and are under intervention to provide the therapeutic relevance of triptolide (Figure 2). Various cellular pathways and signaling cascades were investigated to fine-tune the role of triptolide in bone destruction and reabsorption as active herbal pharmaceutical agents (Chen et al., 2018). Corresponding to conventional traditional medicine relevance, triptolide has shown to exhibit analgesic effects in bone cancer pain relief in rats. Mechanistically, triptolide inhibits the upregulation of HDACs in spinal glial cells to eliminate bone cancer–generated pain (Hu et al., 2017). Osteosarcoma (OS), the most common primary bone tumor that affects adolescents and young adults, is considered the most frequent cause of cancer-related morbidity and mortality. Surgery and chemotherapy are the standard approaches for the treatment of the disease; however, the overall survival remains unsatisfactory due to a high metastasis rate (Simpson and Brown, 2018; Gang et al., 2020). Various studies have demonstrated the potential role of triptolide in the treatment of osteosarcoma. Zhao et al. have shown that triptolide treatment significantly decreases the viability of osteosarcoma cells *in vitro* by decreasing the expression levels of MKP-1 and Hsp70 following treatment (Zhao et al., 2016). In another study, triptolide was shown to inhibit the cell growth and invasion of osteosarcoma by modulating the activity of microRNA-181a, one of the epigenetic factors, via targeting the PTEN gene in the host (Jiang et al., 2017a). AMD3100, the CXCR4 antagonist, in combination with triptolide, induces apoptosis and inhibition of proliferation and invasion of osteosarcoma U2OS cells through regulating the ERK1/2, AKT, and STAT3 pathway and NF-kB activation. In addition, AMD3100 and triptolide treatment reduce the primary growth and lung metastasis of U2OS *in vivo* (Jiang et al., 2017b). Triptolide has been shown to improve the efficacy of cisplatin in both drug-sensitive and drug-resistant osteosarcoma cells, which indicates an interesting therapeutic potential of triptolide for the treatment of osteosarcoma in patients who have reduced responsiveness to cisplatin (Fanelli et al., 2020).

One of the significant causes of bone annihilation is inflammation of and around the bone tissues. A myriad of innate immune signaling cascades and key immune-inflammatory factors such as interferons, cytokines, interleukins, chemokines, etc., were encountered by triptolide to combat bone-related dysfunctions such as osteoclastogenesis, cartilage destruction, and attenuating regional osteoporosis (Yuan et al., 2019). Recent discoveries have identified that triptolide inhibits the PI3K-AKT-NFATc1 pathway and suppresses osteoclastogenesis to prevent bone loss (Cui et al., 2020). A cohort-based study in old male rats showed triptolide supplementation effects on bone microstructure and bone remodeling in the rat lumbar. It is demonstrated that triptolide reduced the expression of the receptor for the activation of the NF-κB ligand (RANKL) and increased osteoprotegerin (OPG) expression in the lumbar. The study proposes the therapeutic usefulness of triptolide for senile osteoporosis as it has a protective effect on age-related bone defects (Luo et al., 2018). *In-vitro* studies demonstrate that triptolide is responsible for suppressing osteoclast differentiation and bone resorption by increasing the production of IL-10 and TGF-β1 through immune regulatory T cells (Xu et al., 2016). In the mouse model, triptolide encounters bone defects by RANKL-mediated NF-қB activation and titanium particle–induced osteolysis (Huang et al., 2015). Triptolide suppresses TNF-α–associated osteoblast differentiation by inhibiting the phosphorylation of nuclear factor-κB in the NF-κB signaling pathway, suggesting the positive effect of triptolide treatment on bone remodeling and fracture repairing (Liu et al., 2017). Numerous advancements have been reported by identifying the role of triptolide in the field of autoimmune-associated bone disorder, rheumatoid arthritis (RA). RA is a disease of joints defined by the inflammation of synovial joints, causing prominent destruction of articular cartilages and erosion of bones. Antirheumatic properties of triptolide have been delineated in different types of cells, such as fibroblast-like synoviocytes (FLSs), T cells, macrophages, dendritic cells, osteoclasts, and chondrocytes. These cells play critical roles in the pathogenesis of RA (Yuan et al., 2019). In addition, triptolide was reported to target the RANKL/RANK/OPG signaling pathway and prevent bone destruction in the collagen-induced arthritis model of RA (Liu et al., 2013). Importantly, triptolide portrays its medicinal and pharmaceutical activity by altering the key immune functions in RA pathogenesis, for example, by regulating the immune-related cells, inflammatory mediators, angiogenesis, bone homeostasis, and cell proliferation (Fan et al., 2018). These studies clearly expand the overall paradigm from basic research to translational clinical adaptation in the pathology of RA, one of the leading bone-related injuries.

The bone marrow (BM) niche plays an important role at different stages of differentiation, migration, proliferation, and drug resistance of malignant plasma cells. An association of BM niche with progression and metastasis to further BM locations is well established in multiple myeloma (MM) (Wallace et al., 2001). Triptolide directly induces apoptosis in bone marrow–derived mesenchymal stem cells (BMMSCs) isolated from patients with MM. In addition, triptolide indirectly affected the proliferation of MM cells by inhibiting IL-6, IL-1β, and stem cell factor (SCF) expression and vascular endothelial growth factor (VEGF) secretion and was suggested to possess a potential anti-MM effect (Wu et al., 2019). As far as molecular mechanism is concerned, triptolide induces apoptosis of multiple myeloma cells through the PI3K/Akt and NF-κB pathway and the MAPK pathway via mitochondrial cell death signaling and caspase activation (Nakazato et al., 2014). Triptolide has the potential as a novel therapeutic agent to treat myeloma. However, more studies are warranted to evaluate this possibility.

## Administration Route of Triptolide

The route of drug administration is always an important topic to understand the mechanism and stability of the drug. The oral route for drug delivery is more common and acceptable, especially for molecules which are difficult to absorb (Xi et al., 2017). Studies have demonstrated that when triptolide is administered through an oral route, it showed more adverse effects (gastrointestinal and liver enzyme changes, among others) than administration through other routes. However, the same study suggested that intravenous administration of triptolide is a better route of delivery because this route overcomes the difficulties of poor solubility and poor absorption (Reinholz et al., 2018; Viegas et al., 2020). The topical route is another method of application that is noninvasive with minimal systemic effects as this route of drug administration avoids the metabolism (Reinholz et al., 2018) (https://doi.org/10.1016/j.nancom.2017.01.003). Several systemic toxicity effects of triptolide on multiple tissues and organs in different animal models and clinical trials have been discussed. Pathological changes after oral and intraperitoneal triptolide administration are observed in lymphatic, gastrointestinal, and reproductive systems, therefore suggesting systemic toxicity (Tao and Lipsky, 2000). Xie et al. have demonstrated a potential mechanism of triptolide-induced nephrotoxicity (Xie et al., 2020a). In a recent study, a triptolide phospholipid complex (TPCX) has been delivered transdermally for the treatment of rheumatoid arthritis (RA) in an animal model (Liu et al., 2021). This study demonstrated that TPCX increased the bioavailability and anti-RA effect of triptolide by increasing its hydrophilicity and transdermal permeability, and therefore, the authors have suggested the great potential of transdermal TPCX cream as a promising approach for the efficient treatment of RA.

## Clinical Potential—From Bench to Bedside

Despite the proven potential of triptolide for various diseases through *in vitro* and *in vivo* studies, there are many challenges to overcome before it moves toward the clinical paradigm. The major challenges are 1) poor water solubility, 2) narrow therapeutic window, 3) multiorgan toxicity, and 4) serious adverse events after long-term usage (Hou et al., 2019; Tong et al., 2021). Researchers are trying to address these challenges by finding triptolide analogs which are easy to deliver, have lower toxicity, and consist of drug-like properties either by synthetic generation or by structural modification.

**FIGURE 3:**
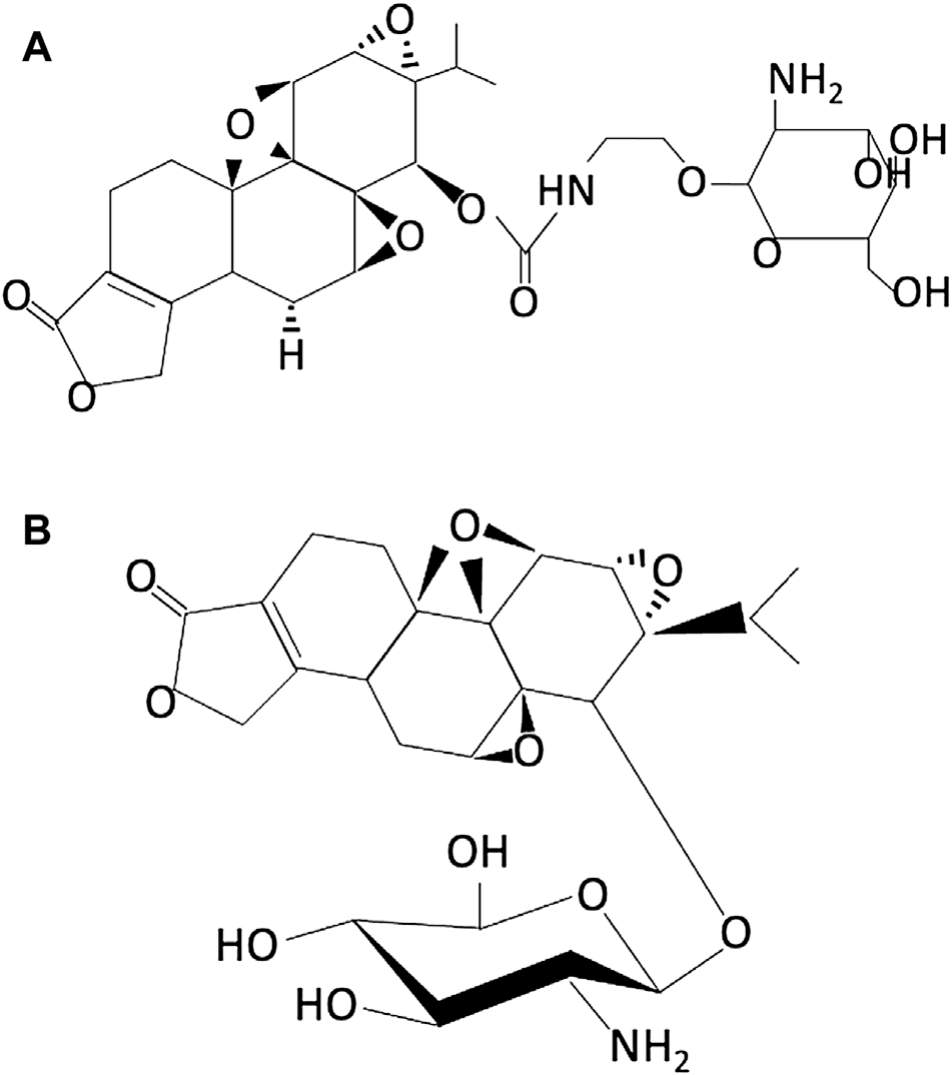
Chemical structure of **(A)** TPG12 (reproduced from Ref 53) and **(B)** TPAG16 (reproduced from Ref 52).

The importance of sugar–protein interactions in lowering the toxicity of drugs and in the delivery of drugs is well studied. Conjugation of small drug molecules to glucose is one of the most useful modes to reduce toxicity and has been reported to improve targeted drug delivery. On similar lines, triptolide glucosamine conjugate products are developed and tested in research studies (Qi et al., 2015; Zhou et al., 2014). Zhou et al. designed and synthesized a triptolide glucosamine conjugate TPG 12 via a carbamate linkage which has low toxicity and high renal uptake (Figure 3A) (Zhou et al., 2014). *In vivo* and *in vitro* results demonstrated that compared to parental triptolide, TPG12 has a significant protective effect against renal ischemia-reperfusion (I/R) injury in rats and has less cytotoxicity, hepatotoxicity, and immunotoxicity. However, the presence of a hydrolytic carbamate linkage in TPG12 makes it biologically unstable and therefore be quickly metabolized by certain esterase *in vivo*. In addition, this modification is found to be neurotoxic in mammals (Chang et al., 2006). To overcome this, Qi and coworkers synthesized a triptolide-2-glucosamine conjugate TPAG 16 via an O-glucosidic bond (TPAG 16) (Figure 3B) (Qi et al., 2015). The glucosamine conjugate (TPAG 16) clearly showed an increased protective effect against renal ischemia/reperfusion (IR) injury and less cytotoxicity, hepatotoxicity, and immunotoxicity than TPG12. However, further studies are required to refine these modifications. Due to the high demand for glucose for update, survival, and rapid growth, cancer cells usually overexpress a series of glucose transporters (GLUTs), particularly GLUT1 and GLUT3 (Macheda et al., 2005). A series of C14 hydroxyl group–modified triptolide glucose conjugates, namely, glutriptolide, have been studied as an effective strategy for tumor-targeted drug delivery with reduced toxicity. He et al. demonstrated that glutriptolide 19 has a higher *in vitro* cytotoxicity against tumor cells over normal cells and better water solubility than triptolide (He et al., 2016). Authors have concluded glutriptolide 19 as a potent and mechanistically novel triptolide prodrug that exerts its anticancer activity through a combination of glucose transporter-based and tumor-targeting activities and inhibition of RNAPII-mediated transcription. Glutriptolide 19 might serve as a promising lead for further development of anticancer drugs.

Apart from various modifications in the molecule itself, associated drug delivery approaches need further intervention. Cell-penetrating peptides (CPPs) are valuable vehicles that could deliver various kinds of therapeutic molecules such as small molecular drugs, nucleic acids, polymers, nanoparticles, etc., across the biomembrane (Habault and Poyet, 2019; Xie et al., 2020b). CPPs, such as polyarginine, TAT, Anp, and PEP-1, are a new class of drug vehicles that could enhance transdermal delivery of small molecules without skin irritation. Because of their positively charged peptides, the polyarginine chain is one of the key elements of the most widely used CPPs as they could interact with cellular membrane components and help in penetration. Tian et al. designed and synthesized a water-soluble triptolide-CPP conjugate TP-disulfide- CR7 (TP-S-S-CR7) 32 which efficiently crosses the barrier of the corneum and reaches the epidermis and dermis after 2 h of transdermal administration. Moreover, TP-S-S-CR7 32 has low cytotoxicity (IC50: 9.06 mM) compared with triptolide (IC50: 1.00 mM) on immortal human keratinocyte cells (Tian et al., 2018; Xu and Liu, 2019). Liu and coworkers demonstrated in their *in vitro* and *in vivo* outcomes that triptolide-nucleic acid aptamer conjugate 33 exhibits good water solubility, high bioavailability, and good targeting and can effectively inhibit the xenograft tumors with fewer side effects. Nanoparticles as a drug delivery method have many advantages including enhanced ability to target cells or tissues, intracellular delivery which in turn helps in overcoming the drug resistance, and controlled release of drugs. Precise delivery of drugs to the target also affects the pharmacokinetic and toxicity profiles of parenteral drugs, enables drug-specific accumulation in the tumor tissue, and releases drugs at a synchronized rate, thereby maintaining synergistic drug ratios to achieve enhanced antitumor effects (Pedziwiatr-Werbicka et al., 2021). A triptolide-loaded silk fibroin nanoparticle (TPL-SFNPs) developed by Ding et al. demonstrated to have a sustained pH-sensitive drug release profile along with biocompatibility characteristics (Ding et al., 2017). Compared with free triptolide, TPL-SFNPs demonstrated enhanced antitumor activity, increased apoptosis of cancer cells, and higher inhibition of colony formation.

Researchers are focusing on the targeted delivery of triptolide to avoid the toxic effects of the drug to normal cells/tissues. Ling and coworkers designed and synthesized a pH-sensitive, folate ligand–attached nanoparticle-encapsulated triptolide (Nf-trip) to treat the folate receptor–overexpressed HCC subpopulation (Ling et al., 2014). Intravenous administration of Nf-trip has demonstrated an accumulation of drugs in the tumor tissue. The pH-sensitive peptide enables the Nf-trip response to the acidic tumor microenvironment and therefore increases the site-specific triptolide release and cell-specific drug uptake which ultimately increased the efficacy and reduced the toxicity. In another study, using the solid dispersion technique, Wang et al. (2016) prepared a conjugated AS1411 aptamer to HOOC-PEG-PDLLA micelle-loaded triptolide AS-PPT, which was tested in chemo-resistant pancreatic cancer (CPC) cell lines (Wang et al., 2016). *In vitro* results demonstrated that AS-PPT had more potent antitumor activity than parental triptolide. Furthermore, AS-PPT possessed a comparable specific binding ability with gemcitabine-resistant human pancreatic cancer cells (MIA PaCa-2). Selective targeting of tumor tissues by AS-PPT was demonstrated using biophotonic imaging.

Minnelide, a water-soluble prodrug of triptolide, was demonstrated to possess antitumor activity in different preclinical cancer models. This prodrug is in phase II clinical trials for advanced refractory adenosquamous carcinoma of the pancreas (ASCP) (https://clinicaltrials.gov/ct2/show/NCT04896073). Another water-soluble analog of triptolide F6008 (PG490-88) has also demonstrated positive results in preclinical mouse xenograft models of lung and colon cancer. Recently, PG490-88 has been shown to have the potential as a prophylactic agent to prevent I/R-induced lung injury (Pao et al., 2019). MRx102, another triptolide derivative, is cytotoxic *in vitro* in human leukemia cells (Reno et al., 2016). These studies demonstrate that triptolide holds the promise to reach its therapeutic potential against various diseases from the laboratory to the clinics in the near future.

## Consequences and Limitations

There are both consequences and limitations of adopting triptolide as a legitimate pharmaceutical drug for conventional regimens and therapy. Meanwhile, triptolide holds the crucial pharmaceutical activity and therapeutic potential to treat a plethora of diseases, ranging from malignancies leading to cancer and anti-inflammation to immunosuppressive properties leading to inflammatory and bone disorders. Some key studies on triptolide in the context of inflammatory and bone-related disorders demonstrate that this drug reduces the production of inflammatory determinants such as TNF-α, IL-6, and IL-17. It alleviates the oxidative stress of damaged tissues and inhibits the activity of immune-inflammatory cells such as T cells and macrophages mainly by exploiting the NF-κB and MAPK signaling pathways of the cellular machinery. The urgency to produce an improved/upgraded version of any multifunctional traditionally acclaimed medicine/drug is always a challenging task in the field of pharmaceutical and health sciences. Despite the great remedial potential, there exist several roadblocks in the procedure of developing and considering triptolide as a new drug/drug candidate. There are several studies on elementary animal models and cell lines deciphering the medicinal importance of triptolide. However, there is still a shortage of data from the clinical trials and the randomized controls cohort-based studies which have direct implications on the usage of the drug by a patient or in a health care system for a particular class of disease. Systemic evaluation of *in vivo* activities of this active compound needs further validation. Triptolide-related toxic parameters and cytotoxicity in healthy tissues and organs need more investigation to eradicate severe side effects of the drug candidate.

## Future Prospects

Future studies focusing on the interaction of triptolide with other drugs would hold potential interest in understanding the mechanism of action using animal models. The synthetic biology principles can be exploited to design, modify, and generate potential therapeutic triptolide derivative(s). Optimizing the compound to highlight the pharmacokinetics and dosage efficacy of the drug through rigorous preclinical *in vivo* and patient-related studies will lead toward improved and accessible outcomes in the health care medical system. There is still a need for more elusive studies on the bioavailability and pharmacodynamics- related data of the medicinal extracts of triptolide that would help interpret the likeliness of triptolide as a pharmaceutical agent. Considering the promising findings, further research is required to investigate the mechanistic modes of action of triptolide using multiomics-based technologies including transcriptomics, proteomics, and metabolomics. In addition, elucidating the pharmacological effects of triptolide in bone-related diseases using cutting-edge approaches such as network pharmacology and molecular docking in the near future would be compelling. Such studies would help discover new drugs, predict the interrelationship network between drugs and diseases, and examine the various pharmacological outcomes.
